# Combined effect of different factors on weight status and cardiometabolic risk in Italian adolescents

**DOI:** 10.1186/s13052-019-0619-9

**Published:** 2019-03-05

**Authors:** Antonino Bianco, Anna Rita Filippi, João Breda, Vincenza Leonardi, Antonio Paoli, Luca Petrigna, Antonio Palma, Garden Tabacchi

**Affiliations:** 1 Sport and Exercise Sciences Research Unit, University of Palermo, Via G. Pascoli 6, 90144 Palermo, Italy; 2 Department of Sciences for Health Promotion and Mother-Child Care “G. D’Alessandro”, University of Palermo, Via Del Vespro 133, 90127 Palermo, Italy; 3 WHO European Office for the Prevention and Control of Noncommunicable Diseases (NCD Office, 9 Leontyevsky Pereulok, Moscow, Russian Federation 125009; 4 Department of Biomedical Sciences, University of Padova, Via Ugo Bassi, 58/B, 35131 Padova, Italy; 5 PhD Program in Health Promotion and Cognitive Sciences, Department of Psychology, Educational Science and Human Movement, University of Palermo, Via G. Pascoli 6, 90144 Palermo, Italy

**Keywords:** Weight status, Cardiometabolic risk, Determinants, Adolescent, Multiple Correspondence Analysis

## Abstract

**Background:**

The observed increase in body weight and cardiometabolic risk (CR) in youth from developed countries contributes to the global burden of chronic diseases in adult age. The aim of this work is to provide a patterning of the associations between different factors and the weight status and CR of the subjects involved in the Italian ministerial ASSO project.

**Methods:**

This study involved 919 students from high schools in Palermo. Weight, height and waist circumference were collected by trained teachers; weight status was estimated by the BMI cut-offs for adolescents and CR through the waist-to-height ratio. Questionnaires were administered through the web-based ASSO-NutFit software. Chi-square test investigated the variables significantly associated with the outcomes, which were then included in a Multiple Correspondence Analysis (MCA), to explore their dimensional relationship to weight status and CR. Poisson regressions were conducted separately for the two outcomes, reporting raw and adjusted prevalence ratios (PRs) and Bootstrap Method was used to determine confidence intervals (CIs), to assessing the degree of effect of the explanatory variables over the outcomes.

**Results:**

Two main dimensions were evidenced, with the overweight/obese group and the group at CR characterized by the following strongly associated factors: male gender, overweight/obese parents, following a slimming regime, caesarean birth, sedentariness, being under/overweight at birth, presence of metabolic risk, going to school by car/scooter, not using supplements.

**Conclusions:**

This study contributed to identifying those adolescents that should be prioritized in interventions aiming at reducing overweight/obesity and CR in this age group.

**Electronic supplementary material:**

The online version of this article (10.1186/s13052-019-0619-9) contains supplementary material, which is available to authorized users.

## Background

The obesity and overweight status in youth have been widely investigated in the last decades and its contribution to the global burden of chronic disease and disability has been highlighted [1, 2]. Children and adolescents classified as overweight or obese exhibit an increase in cardiovascular and metabolic risk factors when compared with those with normal weight [3], this places them at increased risk for many serious health conditions [4]. Changes in BMI and central adiposity in youth positively correlate with changes in cardiometabolic risk (CR) factors [5, 6]. Waist-to-Height Ratio has been proposed as an easily measurable anthropometric index for detection of central obesity and related adverse CR among adults and children [7], but also helps to identify those without central obesity and a healthy risk factor profiles among the overweight/obese children [8, 9].

In 2008 Wang et al. highlighted the increasing interest in understanding the potential link between obesity risk factors and tailoring interventions for different population subgroups [10], especially in youth. Targeted interventions could be translated into the improving of population health and reduction of economical public expenses. In the light of these considerations, some systems addressed to the nutritional and lifestyles surveillance have been developed worldwide [11, 12]. Unfortunately, few data are available in Southern Italy with regard to patterns of obesity and cardiovascular risk factors. The recently piloted Italian ASSO (Adolescents and Surveillance System for the Obesity prevention) Project, funded by the Italian Ministry of Health, aimed to develop and test an innovative web-based system that allows a continuous and standardized data collection about health and lifestyle of adolescents in the school environment [13]. The surveillance system developed by ASSO could be adopted within the National Health Service, this having a big impact on the improvement of the surveillance and prevention efficacy on the national territory [14]. A wide variety of data have been collected through this system, including directly measured anthropometric parameters and socio-demographic, early life, clinical, lifestyle and food habits information, that has been analysed in the present paper. The aims of this work were to evaluate associations between these variables and overweight/obesity risk and CR of the subjects involved in the ministerial ASSO project, and identify groups at risk and patterns of associations by exploring analytically and visually the dimensional relationships.

## Methods

### Participants and data collection

A total of 1021 students were recruited overall (see the sampling procedure in Additional file 1), and among these, 919 delivered consents signed by their parents. All participants were provided with information sheets and had to supply the informed consent signed by their parents.

All data were collected in the school year 2012/2013. Weight, height and waist circumference were collected by the teachers through the use of a calibrated scale, a stadiometer and a non-elastic meter respectively, all available within the schools. Personal, health and lifestyle information were obtained through the administration of three web-based questionnaires included in the ASSO-NutFit software: ASSO-PIQ (Personal Information Questionnaire), ASSO-PASAQ (Physical Activity, Smoke and Alcohol Questionnaire) and ASSO-FHQ (Food Habits Questionnaire). The ASSO-PIQ included questions regarding participant and family information, neonatal and clinical assessment. The ASSO-PASAQ consisted of three sections: physical activity, smoking, alcoholic drinks and other beverages. Finally, the ASSO-FHQ consisted of six items regarding: breakfast, school break, lunch, afternoon break, dinner, and various habits. Students were asked to compile these web-based questionnaires at school, carefully assisted by teachers. For some questions they were alerted the day before, in order to ask some information to their parents (e.g. questions on their breastfeeding, weaning, anthropometric data of parents, etc.). A proper toolkit including the Standard Operating Procedures (SOPs) developed by the ASSO team and all required materials have been provided to the teachers. Afterward, a dedicated training course was administered in order to implement the project correctly and to collect the data properly.

### Variables analysed

The two dependent variables were weight status and CR. Underweight, normal weight, overweight and obese classes were defined using the threshold values for BMI recommended by the International Obesity Task Force [15]. In addition, waist circumference was used to evaluate the waist-to-height ratio, with a cut-off of 0.5 indicating the threshold for subjects at CR [16]. These variables were respectively dichotomized in “under weight/normal weight” - “overweight/obese”, and “not at risk” - “at risk” subjects [16].

The categorical independent variables considered for the association analysis were also dichotomized and grouped as in Table 1 (details on the variables characteristics are explained in the table).

**Table 1:** Sample composition by socio-demographic, early, clinical, lifestyle factors and food habits of the ASSO subjects

Parameter 1	Parameter 2	N (%); *(father) (%), [mother] [%]*
*Socio-demographic factors*		
Gender***^$$$^	Male	524 (60.7)
	Female	339 (39.3)
Age	14–15 years	355 (41.1)
	16–17 years	508 (58.9)
Marital status	Married	1204 (85.9); *(601) (86.1), [603] [85.7]*
	Other	198 (14.1); *(97) (13.9), [101] [14.3]*
Weight status***^$^	Normal weight	758 (54.1); *(279) (40.1), [479] [68.2]*
	Overweight/obese	640 (45.8); *(417) (59.9), [223] [31.8]*
Education	Low	407 (29); (206) (29.5)***^$^, [201] [28.6]*^$$^
	High	995 (70.9); (492) (70.5)***^$^, [503] [71.4]*^$$^
Occupation	Occupied	1030 (73.5); *(621) (89.0), [409] [58.1]*
	Other	372 (26.45); *(77) (11.0), [295] [41.9]*
Profession	Manual job	118 (10.5); *(94) (15.1),* [24] *[5.9]*
	Non-manual job	912 (89.5); *(527) (84.9), [385] [94.1]*
Family Affluence Scale^a^	Medium/low	309 (43.6)
	High	400 (56.4)
Pocket money	Up to € 10	659 (92.9)
	More than € 10	50 (7.1)
*Early life factors*		
Term of birth	Full-term	588 (82.9)
	Pre-term	121 (17.1)
Breastfeeding	Yes	569 (80.3)
	Not	140 (19.7)
Birth weight**	Normal	464 (65.4)
	Under/over weight	245 (34.6)
Breastfeeding duration	3–5 months	466 (81.9)
	6 months	103 (18.1)
Type of birth*	Natural	376 (53.0)
	Caesarean	333 (47.0)
Weaning	3–5 months	533 (75.2)
	6 months	176 (24.8)
*Clinical aspects*		
Diagnosed diseases^b^	Not	617 (87.0)
	Yes	92 (13.0)
Family diseases^b^	Not	284 (40.1)
Use of drugs	Not	663 (93.5)
	Yes	46 (6.5)
Psycho-physical malaise score^c^	Low/very low	486 (68.5)
	High/very high	223 (31.5)
*Lifestyles*		
Use of supplements^d^**^$^	Not	641 (90.4)
	Yes	68 (9.6)
Smoking frequency	Lower than 7 days/week	25 (40.3)
	7 days/week	37 (59.7)
Slimming regime***^$$$^	Not	585 (82.5)
	Yes	124 (17.5)
Alcohol consumer	Not	195 (37.5)
	Yes/sometimes	325 (62.5)
Way to go to school^$^	Walking/by bike	126 (24.2)
	By car/scooter	394 (75.8)
Type of alcoholic drinks	Beer/wine/light drinks	198 (60.9)
	Spirits	127 (39.1)
Physical activity status^e^*^$^	Active	325 (62.5)
	Sedentary	195 (37.5)
Alcoholic risk^f^	Not at risk	467 (89.8)
	At risk	53 (10.2)
Smoke	Non-smoker/ experimenter^g^	458 (88.1)
	Sporadic/weekly/daily^h^	62 (11.9)
Coffee consumption	Not	296 (56.9)
	Yes	224 (43.1)
*Food habits*		
Breakfast consumption	Regular	457 (62.6)
	None/sporadic	273 (37.4)
Between meals	None	337 (46.2)
	At least one	393 (53.8)
Breakfast adequacy^i^	Adequate	226 (31.0)
	Inadequate	504 (69.0)
Industrial foods consumption	Up to one/week	546 (74.8)
	More than one/week	184 (25.2)
School-break adequacy^i^	Adequate	176 (24.1)
	Inadequate	554 (75.9)
Organic food consumption	Yes	391 (53.6)
	Not	339 (46.4)
Lunch adequacy^l^	Adequate	549 (75.2)
	Inadequate	181 (24.8)
Fresh vegetables/fruit consumption	Yes	560 (76.7)
	Not	170 (23.3)
Afternoon-break adequacy^i^	Adequate	204 (27.9)
	Inadequate	526 (72.1)
Vending machines foods consumption	Not	375 (51.4)
	Yes	355 (48.6)
Dinner adequacy^l^	Adequate	489 (67.0)
	Inadequate	241 (33.0)

*p < 0.05, **p < 0.01, ***p < 0.001. Significant associations with weight status, estimated by a chi-square test ^$^*p* < 0.05, ^$$^*p* < 0.01, ^$$$^*p* < 0.001. Significant associations with CR, estimated by a chi-square test,

^a^The Family Affluence Scale (FAS) is a scale constructed by the sum of scores of the answers to questions about the presence of common goods such as computers, number of cars, single bedroom and annual holidays^17^

^b^Obesity, cardiovascular diseases, high triglycerides/cholesterol, diabetes, hypertension, liver diseases, osteoporosis, cancer, food allergies, and food intolerances

^c^The score of psycho-physical malaise was constructed on the presence of one or more symptoms: headache, stomach ache, backache, feeling low, irritability or bad mood, nervousness or anxiety, sleeping difficulties, dizziness [53]

^d^Vitamins; mixed minerals, single minerals such as iron or calcium; proteins, aminoacids, creatin carnitin; fibers; other

^e^Assessed by considering “sedentary” whoever stayed on average more than 3 h a day watching TV, playing computers or videogames

^f^Calculated on the basis of alcoholic daily intake greater than 12 g. It is identified as the risk of health problems related to alcohol abuse

^g^Non smoker: never tried smoking in his/her life; Experimenter: tried smoking at least once in his/her life

^h^Sporadic: smokes less than one cigarette per week

^i^Evaluated with resoect to a proper meal excluding carbonated beverages or other junk food

^l^Evaluated with respect to a proper meal including a first or a second course with vegetables, fruit, bread, and excluding carbonated beverages or other junk food

### Statistical analysis

Recorded information was automatically converted by the software into an excel database, that was analysed using the statistical software STATA/MP 12.1 (StataCorpLP, college Station, TX, USA).

Frequencies were reported on categorical variables. Pearson’s Chi-square test investigated the variables significantly associated with the outcomes.

Variables found to be associated with the two outcomes were included in a Multiple Correspondence Analysis (MCA), a suitable method for population-based studies that projects data into space-dimensions and searches for patterns in the dataset, helping to visualize the variables more closely associated with different groups [17]. The number of dimensions was chosen by analysing the decline of eigenvalues. The outcome variables were included as supplementary points in the analysis.

Finally, the degree of effect of the explanatory variables over the outcomes was assessed through the estimation of Prevalence Ratios (PRs). In analyses of data from cross-sectional studies, when the binary outcome is common and usually with a prevalence greater than 10%, PRs are more appropriate than Odds Ratios as they can be overestimated by the OR and can be better controlled for confounding [18–20]. Moreover, the Cox and Poisson models with robust variance are better alternatives than logistic regression in order to estimate PRs [21]. Therefore, raw and adjusted PRs were estimated using the Poisson regression model, and their 95% confidence intervals (CI) were obtained using Bootstrap Method.

All statistical analyses were performed considering an alpha level of 0.05 as significant.

## Results

### Sample composition

The sample composition by socio-demographic factors, early life factors, clinical aspects, lifestyles and food habits, is shown in Table 1. Gender, weight status and education of the parents, birth weight and type of birth, use of supplements, slimming regime, way to go to school and physical activity status, were found to be associated with the outcomes (Table 1) and, then, entered the MCA.

Weight status was evaluated on 759 subjects, this accounting for the 83% of the subjects involved; the other 17% were not present at school the day of collection, or were ashamed to put off their shoes for the weight and height collection by the teachers. More than a quarter of adolescents were overweight/obese; a significantly higher proportion of males were overweight/obese (30.2%) compared to females (16.2%) (*p* < 0.001) (Table 2), with a PR of 1.54 (CI 1.09–2.10) for males compared to females (Table 3).

**Table 2:** Weight status and cardiometabolic risk in the total sample and in the sample stratified by gender of the adolescents participating in the ASSO project

	Total		Males		Females	
	n	%	n	%	n	%
*Weight status*						
Under weight	6	0.8	3	0.6	3	0.9
Normal weight	540	71.1	295	56.3	245	72.3
Over weight	172	22.7	123	23.5	49	14.5
Obese	41	5.4	35	6.7	6	1.8
Under + Normal weight	546	71.9	298	56.9	248	73.2
Over weight + Obese***	213	28.1	158	30.2	55	16.2
Total^a^	*759*	*100*	*456*	*60.1*	*303*	*39.9*
*Cardiometabolic Risk*						
Non at risk	511	72.8	278	65.9	233	83.2
At risk***	191	27.2	144	34.1	47	16.8
Total^a^	*702*	*100*	*422*	*60.1*	*280*	*39.9*

***p < 0.001. Significant difference between males and females, estimated by a chi-square test

^a^Percentages are referred to the total sample

**Table 3:** Univariate and multivariate analysis of the associations between weight status/cardiometabolic risk (CR) and selected variables

	Weight status		CR	
Variables	*Raw PR* ^*a*^ *(95% CI)*	*Adj PR (95% CI)*	*Raw PR (95% CI)*	*Adj PR (95% CI)*
Gender (Male vs Female)	1.91 (1.31–2.66)	1.54 (1.09–2.10)	2.03 (1.46–2.83)	1.91 (1.24–2.91)
Father weight status (Overweight/Obese vs Normal)	1.75 (1.23–2.21)	1.57 (1.20–2.28)	NS	NS
Father education (High vs Low)	0.62 (0.45–0.77)	NS	NS	NS
Mother weight status (Overweight/Obese vs Normal)	1.78 (1.34–2.22)	1.56 (1.04–2.08)	1.50 (1.10–2.04)	NS
Mother education (High vs Low)	0.72 (0.54–0.90)	NS	0.67 (0.49–0.93)	NS
Birth weight (Under/Overweight vs Normal)	1.51 (1.05–1.95)	1.46 (1.10–1.99)	NS	NS
Birth type (Caesarean vs Natural)	1.34 (1.02–1.63)	1.39 (1.03–2.02)	NS	NS
Use of supplements (Yes vs Not)	0.43 (0.18–0.71)	0.39 (0.10–0.68)	NS	NS
Slimming regime (Yes vs Not)	2.50 (1.81–3.11)	2.11 (1.52–2.79)	2.52 (1.83–3.46)	2.09 (1.41–3.11)
Way to go to school (By car/scooter vs Walking/by bike)	NS	NS	1.68 (1.05–2.70)	NS
Physical activity status (Sedentary vs Active)	1.43 (1.01–1.81)	NS	2.16 (1.46–3.17)	NS

^a^Raw and adjusted prevalence ratio (PR) with 95% confidence interval (CI) performed through Poisson regression of the subjects’ weight status and cardiometabolic risk (CR)

CR was evaluated on 702 subjects; the other participants were not present at school the day of collection, or were ashamed to show their belly and refused to be measured. Among these, 27.2% were at CR. (Table 2). Males had higher prevalence of CR compared to females (Adj PR 2.03, CI 1.24–2.91) (Table 3). Weight status and CR were significantly associated, with overweight/obese subjects being at higher CR (PR 7.86, CI 5.68–10.87, p < 0.001).

### Multiple Correspondence Analysis

The Multiple Correspondence Analysis favoured dimensions 1 and 2 for a total of 73.5% explained Chi-squared distribution. These two orthogonal dimensions explained about 73.5% of general Chi-squared distribution.

**Fig. 1:**
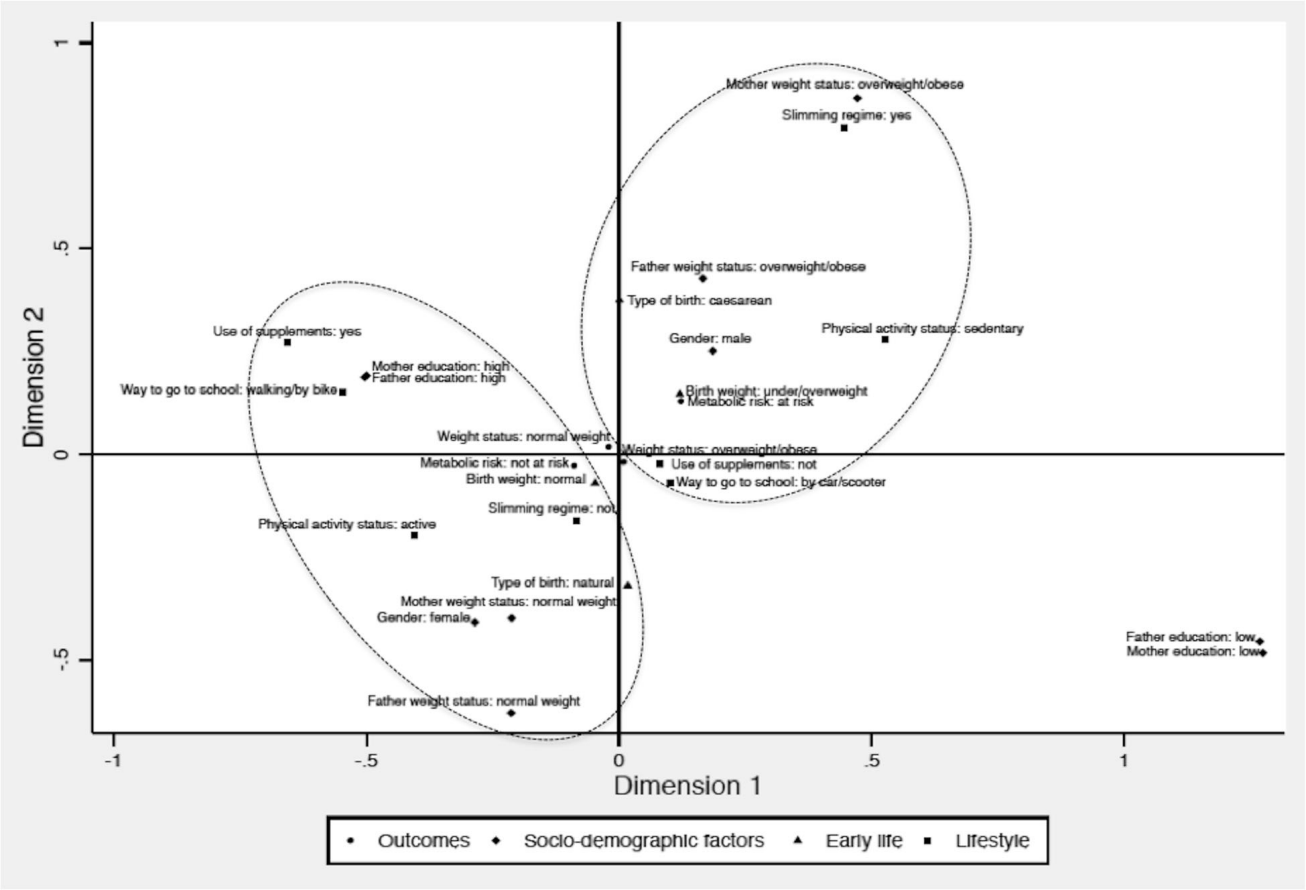
Multiple correspondence analysis of weight status and cardiometabolic risk with all associated variables

Graphically, in the 2-dimensional space, the proximity of categories and their location along the axes (horizontal or vertical for Dimension 1 and Dimension 2, respectively) helps to identify variables that are the most correlated with each dimension. Figure 1 shows lifestyles factors to be the variables contributing to the first dimension (explaining about 40.8%): the categories *being active* vs *sedentary*, *consume supplements* vs *not consume supplements*, *going to school walking/by bike* vs *going to school by car/scooter* are located along the horizontal axis. Dimension 2 consisted of socio-demographic and early factors (explaining about 32.7%): the categories *natural birth* vs *caesarean birth*, *normal weight birth* vs *under- or over-weight birth*, *normal weight parents* vs *overweight or obese parents* are located along the vertical axis (Fig. 1). Then, analysing the pattern of categories around the outcomes (weight status and CR), the following characteristics were observed for the overweight/obese group and the group at CR: male gender, overweight/obese parents, following a slimming regime, caesarean type of birth, sedentary physical activity status, being under/overweight at birth, presence of metabolic risk, going to school by car/scooter, not using supplements (Fig. 1).

### Prevalence ratios

Raw and adjusted PR with 95% CI for weight status and CR are showed in Table 3. After adjusting for confounders, father and mother education were not associated anymore with weight status of their sons, as well as, the physical activity status. In the multivariate analysis, males were around one time and a half more overweight/obese than females (Adj PR 1.54, CI 1.09–2.10), and for adolescents whose father or mother was overweight/obese were one and a half time more overweight/obese compared to adolescents with normal weight parents (Adj PR 1.57, CI 1.20–2.28; Adj PR 1.56, CI 1.04–2.08 respectively). In addition, weight status increased in adolescents being under/overweight at birth (Adj PR 1.46, CI 1.10–1.99) and born with caesarean delivery (Adj PR 1.39, CI 1.03–2.02), while it decreased among supplements consumers (Adj PR 0.39, CI 0.10–0.68); finally, those adolescents following a slimming regime were two times overweight/obese than those who were not at regime (Adj PR 2.11, CI 1.52–2.79).

With regard to the CR, the adjustment for confounding factors removed the association with mother weight status and mother education, and with the way to go to school and sedentariness/activity (Table 3). Males were at CR around twice more compared to females (Adj PR 1.91, CI 1.24–2.91). Slimming regime was an independent factor to be more at CR of around twice (Adj PR 2.09, CI 1.41–3.11).

## Discussion

The present study investigated the characteristics of a sample of adolescents in Southern Italy and the associations of weight status and CR with socio-demographic, early life, clinical, lifestyle and food habits factors. The following variables were found to be significantly associated with the two outcomes: male gender, overweight/obese parents, following a slimming regime, caesarean type of birth, sedentary physical activity status, being under/overweight at birth, presence of metabolic risk, going to school by car/scooter, not using supplements.

The prevalence of overweight/obese adolescents in the analysed sample is well higher than the correspondent prevalence in the HBSC sample of Sicilian adolescents aged 15 (28.1% vs 18.3%) [22]. This difference may be due to the fact that our sample is collected from an urban area of Sicily, which could include adolescents with different lifestyle and behaviours compared to students coming also from rural areas, such as in the HBSC study. It is noticeable that our measures were collected directly on subjects involved, while BMI measure in HBSC study was self-reported.

As regards CR, there are not reference data to carry out comparisons in terms of prevalence, but a high association with the weight status was observed, thus suggesting that preventive actions should also take into account factors related to metabolism and cardiovascular system, and not only to weight status of adolescents.

In line with Bolton et al. [23], weight status was found to be significantly associated with gender, and the association remained even after adjustment for confounders; this suggests that health promotion initiatives could be tailored to be gender specific. Gender patterns may indicate that environmental influences are more detrimental for boys or those preventive interventions are less effective [24].

Our results showed that adolescents whose parents were overweight/obese were twice more overweight/obese and this remained after adjustment for confounding factors. The association between parents’ weight status and their kids’ weight is widely reported in the literature [25–28], and may be due to a combination of genetic and lifestyle drivers [29–34]. Also for the CR associations were found in the bivariate analysis, but the effect disappeared after adjustment.

Significant associations were found between subjects’ weight status and their parents’ education, with a higher education level being protective against the overweight/obesity development. This is in line with the review from Shrewsbury et al. [35], where parental education was inversely associated with children adiposity in most studies, and with the most recent review and meta-analysis stating that parental educational level was consistently inversely associated with childhood overweight and obesity than other indicators [36]. Nevertheless, in our analysis the education was not influencing the overweight/obesity status anymore after adjustment, thus indicating that it is not an independent risk factor. Education is an important socio-economic status indicator, but does not entirely capture the financial aspects of socio-economic status; results of the mentioned meta-analysis state that lower socio economic position had higher risks of overweight and obesity, and the increased risks were independent of the income levels of countries [36]. In our analysis, parents’ occupation, profession and Family Affluence Scale (FAS) that are indicators of the economic aspect of the family, were found not to be associated with the weight status and CR of the adolescents. This finding is not in line with a wide literature stating that subjects from low family SES in industrialized countries are at increased risk of being obese than their counterparts [36, 37].

Regarding the CR, high parents’ education is not a protective factor, as evidenced from the analysis after adjustment.

Among early life factors, birth weight and delivery type showed significant association with weight status; according to another study [38], a child’s risk of future overweight can be determined on the basis of its weight and BMI measures from early infancy, and throughout early childhood with even greater certainty. Results about the association of weight status with the birth delivery was in line with Li et al. [39], observing caesarean section moderately associated with offspring overweight and obesity.

The use of supplements is a strong preventive factor of weight gain in our study. Even though the question concerning supplements was asking for the use of different supplements, it was noticed that students using supplements where mostly those having an active life and practising sport.

Walking or going by bike to school was found to be protective only against CR, even though this effect was not present after adjustment; similar results were observed in Pizarro et al. [40], where exertions to increase and maintain walking to school was observed to likely have a positive impact on children’s health and eventually decrease metabolic and cardiovascular diseases.

A sedentary physical activity status was found to be associated with the increased risk of overweight/obesity and of CR. This is in line with several studies reported in a systematic literature review stating that sedentary behaviour (assessed primarily through increased TV viewing) for more than 2 h per day was associated with unfavourable body composition [41]. However, when analysing the relationship between sedentary behaviour and weight status, few studies control for confounding factors such as diet and physical activity. In our study, in fact, the effect disappeared when adjusting for confounders. This is in line with a meta-analysis of mainly cross-sectional studies, stating that the strength of such an association was actually very small [42].

Sedentariness was also initially associated with the CR, this according to the study from Machado-Rodrigues et al. [43] stating that increased TV viewing had an adverse effect on metabolic health of adolescent girls. After the adjustment for confounders, the association disappeared, and this is in line with the review reporting that, although increased sedentary time was associated with increased health risk, there was insufficient evidence to draw conclusions on the relationship for metabolic risk as a whole [41].

Overall, even though there was not an association between smoke and weight status or CR, the percentage of smokers and the frequency of consumption was high. These data are discouraging, because smoking, especially in adolescence, involves major diseases in the lung that are among the top causes of death in Western countries.

No associations were found with nerve stimulant beverages and alcoholic drinks use. While there is no literature on the effect of nerve stimulant beverages, few studies have explored the association between alcohol use and body weight in adolescence. In some cross-sectional studies overweight/obesity has been shown to be significantly associated with youth substance use, including alcohol [44, 45].

Weight status and CR were found not to be significantly associated with variables relating to food habits and clinical aspects. Regarding dietary behaviours, some studies showed an inconsistent relationship between fruit and vegetable consumption and a child BMI [46, 47], and a negative association between regular breakfast consumption and childhood obesity outcomes [48, 49]. On the contrary, another study suggests that eating breakfast is associated with a reduced risk of becoming overweight or obese and a reduction in the BMI in children and adolescents in Europe [50]. However, breaks include often high-calorie foods such as packaged snacks and unhealthy snacks baked products in our sample. Almost three quarters of students, in fact, don’t have an adequate school or afternoon break. The result on clinical aspects, such as psycho-physical malaise score are not in line with the study of Castro-Pinero et al. [51] stating that overweight-obesity increased the risk of having health complaints in youth.

The Multiple Correspondence Analysis helped identifying groups at higher risk of overweight/obesity and with CR. The overweight/obese group and the group at CR presented the following characteristics: male gender, overweight/obese parents, following a slimming regime, caesarean type of birth, sedentary physical activity status, under/overweight at birth, going to school by car/scooter, not using supplements. The overweight/obese group and the group at CR were closest to the Y axis, and, consequently, more associated with dimension 2, which means that the categories of variables that made up this dimension (socio-demographic and early factors) were the variables that contributed the most to describe this group. As few other studies have been using the MCA, it is difficult to provide comparisons with other authors. E.g. one study found also a dimension made up from socio-demographic, clinical and health behaviors factors on the sitting time, but it was on adult population and in a developing country [52]. The observed results suggest the need of different interventions addressed to adolescents in order to change their sedentary lifestyles, addressed to mothers to change their behaviors in the perinatal period, and targeted in particular to male adolescents with overweight/obese parents.

The strengths of the present study are mainly methodological. The ASSO project, in fact, is aimed at collecting valid and reliable data by using a standardized methodology. Web questionnaires were developed by experts and weight, height and waist circumference were directly measured by trained operators instead of being self-reported. The possibility of collecting such a valid data, provides an added value to this study, as information on the interconnections between different variables in an adolescent population from Southern Italy is presented here, thus adding important and previously missing data to the literature. The use of the MCA, moreover, is particularly relevant in studies where a large amount of qualitative data is collected, such as in the ASSO surveillance system; it is a particularly powerful method as uncovers groupings of variable categories in the dimensional spaces, providing key insights on relationships between categories, without needing to meet assumptions requirements.

One methodological limitation, however, is that an objective method to assess sedentary behavior (e.g. accelerometry) has not been used. A second limitation is the high heterogeneity of the dimension 1 data; maybe due to the fact that are self-reported information coming from adolescents, while the one of dimension 2 (reported by the parents) seem to be more stable. Moreover, the study sample was from a single city, thus reducing the generalizability of the results to a larger population, and was composed of a higher number of male adolescents (65%) compared to females; this was due to the sample stratification per school typology, which did not take into consideration the gender distribution of each school.

## Conclusions

This study identified in some socio-demographic, early factors and lifestyle factors important determinants of overweight/obesity status and cardiometabolic risk in a sample of adolescents from Southern Italy. The evidenced pattern included male gender, caesarean delivery, under/over birth weight, parents’ overweight/obesity, sedentariness, being at metabolic risk and not using supplements. It contributed to identifying those groups of adolescents that should be prioritized in interventions in Southern Italy aiming at reducing overweight/obesity and CR in this age group.

It can be hypothesized that specific prevention actions could be targeted to adolescent males; or they could be focussed on those families (parents) presenting a condition of overweight/obesity, by warning them on the importance of educate their kids to take care about their weight status and their health. Actions should be also reinforced for pregnant women, in order to make them more aware of the significance of a correct birth weight and of the importance of a natural delivery for the child’s health.

## Additional file

Additional file 1: Sampling procedure (DOCX 88 kb)

## Abbreviations

ASSO: Adolescents and Surveillance System for the Obesity prevention
ASSO-FHQ: Food Habits Questionnaire
ASSO-PASAQ: Physical Activity, Smoke and Alcohol Questionnaire
ASSO-PIQ: Personal Information Questionnaire
BMI: Body Mass Index
CI: confidence interval
CR: cardiometabolic risk
FAS: Family Affluence Scale
MCA: Multiple Correspondence Analysis
PR: Prevalence ratio
SOPs: Standard Operating Procedures
