# Supplementary material for: Combined effect of different factors on weight status and cardiometabolic risk in Italian adolescents
Source: Ital J Pediatr. 2019 Mar 5;45:32. doi: 10.1186/s13052-019-0619-9 (PMC6402148; doi:10.1186/s13052-019-0619-9)
Supplement: Supplementary file 1 — Sampling procedure (DOCX 88 kb) [file 13052_2019_619_MOESM1_ESM.docx]

**Additional file 1 - *Sampling procedure***

The selection of subjects participating in the Project consisted of a multistage sampling: a first sample stage included a systematic sampling of the 55 high public and private schools of Palermo, whose list was provided by the Ufficio Scolastico Provinciale; among these schools a stratification per type of school (technical, professional, classic, scientific, artistic) took to the selection of 7 schools; in a second stage, a cluster sampling of the first four classes was performed, in order to obtain subjects in the range of 14-17 years, and excluding class five where 18 years and older subjects were present; in the third stage, all the students in the classes were selected.

For the calculation of the required sample size, the following formula was applied: n = [t² x p (1 - p)] / m², where: n = required sample size; t = confidence level at 95% (standard value of 1.96); p = estimated prevalence of overweight/obesity in the project area (evaluated on an attended frequency of overweight/obese subjects of 25%, in accordance with recent data from the HBSC Study Italy 2011); m = margin of error at 3% (value of 0.03). Then, an oversampling proportional to the questionnaire completion attrition rate (37%) (evaluated on the basis of the same HBSC Study, where the percentage of questionnaires obtained was around 63%), by calculating the non respondents and adding them to the required sample size. The total sample size was then calculated to be 1,096.
